# Bioavailability and toxicity after oral administration of *m*-iodobenzylguanidine (MIBG)

**DOI:** 10.1038/sj.bjc.6690128

**Published:** 1999-02

**Authors:** A Kuin, M Rutgers, M A van der Valk, J H Beijnen, L A Smets

**Affiliations:** Department of Experimental Therapy, The Netherlands Cancer Institute/Antoni van Leeuwenhoek Hospital;, Amsterdam, The Netherlands; Department of Experimental Animal Pathology, The Netherlands Cancer Institute/Antoni van Leeuwenhoek Hospital;, Amsterdam, The Netherlands; Department of Pharmacy and Pharmacology, Slotervaart Hospital/The Netherlands Cancer Institute, Amsterdam, The Netherlands

**Keywords:** MIBG, oral administration, bioavailability, toxicity, carcinoid syndrome

## Abstract

*meta*-iodobenzylguanidine (MIBG) radiolabelled with iodine-131 is used for diagnosis and treatment of neuroadrenergic neoplasms such as phaeochromocytoma and neuroblastoma. In addition, non-radiolabelled MIBG, administered i.v., is used in several clinical studies. These include palliation of the carcinoid syndrome, in which MIBG proved to be effective in 60% of the patients. Oral MIBG administration might be convenient to maintain palliation and possibly improve the percentage of responders. We have, therefore, investigated the feasibility of oral administration of MIBG in an animal model. Orally administered MIBG demonstrated a bioavailability of 59%, with a maximal tolerated dose of 60 mg kg^−1^. The first and only toxicity encountered was a decrease in renal function, measured by a reduced clearance of [^51^Cr]EDTA and accompanied by histological tubular damage. Repeated MIBG administration of 40 mg kg^−1^for 5 sequential days or of 20 mg kg^−1^for two courses of 5 sequential days with a 2-day interval did not affect renal clearance and was not accompanied by histological abnormalities in kidney, stomach, intestines, liver, heart, lungs, thymus, salivary glands and testes. Because of a sufficient bioavailability in absence of gastrointestinal toxicity, MIBG is considered suitable for further clinical investigation of repeated oral administration in patients. *1999 Cancer Research Campaign*


*British Journal of Cancer* (1999) **79**(5/6), 802–806
© 1999 Cancer Research Campaign
Article no. bjoc.1998.0128

### Bioavailability and toxicity after oral administration of *m*-iodobenzylguanidine (MIBG)

**A Kuin[1], M Rutgers[1], MA van der Valk[2], JH Beijnen[3] and LA Smets[1]**

Departments of [1]Experimental Therapy and [2]Experimental Animal Pathology, The Netherlands Cancer Institute/Antoni van Leeuwenhoek Hospital; [3]Department of Pharmacy and Pharmacology, Slotervaart Hospital/The Netherlands Cancer Institute, Amsterdam, The Netherlands

**Summary** *meta*-iodobenzylguanidine (MIBG) radiolabelled with iodine-131 is used for diagnosis and treatment of neuroadrenergic neoplasms such as phaeochromocytoma and neuroblastoma. In addition, non-radiolabelled MIBG, administered i.v., is used in several clinical studies. These include palliation of the carcinoid syndrome, in which MIBG proved to be effective in 60% of the patients. Oral MIBG administration might be convenient to maintain palliation and possibly improve the percentage of responders. We have, therefore, investigated the feasibility of oral administration of MIBG in an animal model. Orally administered MIBG demonstrated a bioavailability of 59%, with a maximal tolerated dose of 60 mg kg$^{-1}$. The first and only toxicity encountered was a decrease in renal function, measured by a reduced clearance of [$^{51}$Cr]EDTA and accompanied by histological tubular damage. Repeated MIBG administration of 40 mg kg$^{-1}$ for 5 sequential days or of 20 mg kg$^{-1}$ for two courses of 5 sequential days with a 2-day interval did not affect renal clearance and was not accompanied by histological abnormalities in kidney, stomach, intestines, liver, heart, lungs, thymus, salivary glands and testes. Because of a sufficient bioavailability in absence of gastrointestinal toxicity, MIBG is considered suitable for further clinical investigation of repeated oral administration in patients.

**Keywords:** MIBG; oral administration; bioavailability; toxicity; carcinoid syndrome

*meta*-iodobenzylguanidine (MIBG) is a multipotent drug which is increasingly applied in clinical oncology. As a derivative of the neuron-blocking agents bretylium and guanethidine, radioiodinated [$^{131}$]MIBG has been primarily developed for the scintigraphic detection of neuroadrenergic tissues (Wieland et al, 1980). Subsequently, [$^{131}$]MIBG has been used at elevated radioactive doses for endo-irradiation of neuroadrenergic neoplasms such as phaeochromocytoma and neuroblastoma (Sisson and Wieland, 1986; Hoefnagel et al, 1987 *a*). The accumulation of MIBG in normal and neoplastic tissues of neuroadrenergic origin is based on its affinity for the specific uptake-1 mechanism of catecholamines (Jaques et al, 1987; Smets et al, 1991). Because of cross-affinity for the serotonin transporter (Rutgers et al, 1993), [$^{131}$I]MIBG is also applied for diagnosis and treatment of carcinoid tumours (Hoefnagel et al, 1987*b*; Taal et al, 1996*a*).

In the course of clinical investigations, non-radiolabelled MIBG is administered as a pre-dose to improve the tumour uptake and retention of a subsequent [$^{131}$I]MIBG dose (Taal et al, 1996*b*; Hoefnagel et al, 1998). Moreover, non-radiolabelled MIBG induces long-lasting palliative responses of the carcinoid syndrome in 60% of the patients, similar to those obtained with targeted radiotherapy with [$^{131}$I]MIBG (Taal et al, 1996*a*). This syndrome, with episodes of diarrhoea, flushing, wheezing, vomiting and/or palpitations, and in several cases the ultimate development of lethal cardiac failure, is due to high production and sudden release of vasoactive compounds, predominantly serotonin, from liver metastases. Despite the subjective improvement, neither tumour regression nor decreases in urinary levels of the serotonin metabolite 5-HIAA were observed. For palliative treatment unlabelled MIBG is preferred over [$^{131}$I]MIBG therapy, especially in elderly patients or patients in poor condition, because no isolation is needed (Taal et al, 1996*a*).

Although the mechanism of palliation by MIBG is unknown, pharmacological effects on serotonin storage or release, or effects at the level of the serotonin 5-HT receptor are most probable. However, contributions of mitochondrial inhibition by MIBG observed in vitro (Loesberg et al, 1990*a*) and in vivo (Kuin et al, 1994), vascular effects by inhibition of nitric oxide synthase (NOS) (Kuin et al, 1998) or interference with mono(ADP)ribosylation (Loesberg et al, 1990*b*), cannot be excluded.

Side-effects attributable to unlabelled MIBG in patients are transient changes in blood pressure and pallor, which are dose-limiting but disappear shortly after the end of the infusion (Taal et al, 1996*a*). In mice, i.v. administered MIBG is acutely lethal at a bolus injection of > 25 mg kg$^{-1}$ (M. Rutgers, unpublished results). After i.p. administration, MIBG appeared to be lethal at single doses of just over 40 mg kg$^{-1}$ MIBG, but not at 40 mg kg$^{-1}$ for 9 sequential days (Smets et al, 1988). Side-effects encountered above 30 mg kg$^{-1}$ i.p. are stress-related, sympathomimetic effects, plausibly explained by the release of bioamines from their storage granules (Kuin et al, 1994), and reduced renal clearance rates accompanied by histological tubular damage (Kuin et al, 1998).

In patients, MIBG administered in an oral formulation might maintain palliation of the carcinoid syndrome without need for hospitalization. Moreover, MIBG might prevent the acute side-effects associated with i.v. infusion, allowing higher doses, which

*Received 30 January 1998*
*Accepted 30 June 1998*

*Correspondence to:* LA Smets, The Netherlands Cancer Institute, Division of Experimental Therapy, 120 Plesmanlaan, NL 1066 CX Amsterdam, The Netherlands may increase the percentage of responders. Because of multiple biochemical effects, gastrointestinal toxicity is of concern with this route of administration. In addition, MIBG, with a lipophilic benzyl group and a positively charged diamine group at physiological pH, is a typical organic cation and thus a potential substrate for P-glycoprotein-mediated export in the gastrointestinal tract. Adequate absorption, therefore, may be limited as demonstrated for paclitaxel (Sparreboom et al, 1997). Moreover, biodistribution of oral MIBG may vary considerably for oral and i.v. routes.

In the present study, we have explored the feasibility of oral administration of MIBG by studying the bioavailability and biodistribution of [$^{125}$I]MIBG in C3H/Km mice. Moreover, renal function and administration route-related toxicity were examined after single and repeated oral administration.

### MATERIALS AND METHODS

### Animals and treatment protocols

The animal experiments were carried out in accordance with protocols approved by the experimental animal welfare committee of the institute and conform to national and European legislation for animal experimentation. In all experiments C3H/Km male mice were used at an age of 10–12 weeks, weighing 28–35 g.

MIBG. $^1/_2$ $H_2SO_4$ (EMKA Chemie, Germany) was dissolved in phosphate-buffered saline (PBS) and administered orally at doses of up to 80 mg kg$^{-1}$ with the aid of a blunt needle. Intravenous injection of 15 mg kg$^{-1}$ MIBG, which appeared to be just below the maximal tolerated dose in this strain of mice, required slow administration over approximately 1 min. For repeated oral administration animals were treated daily with 40 mg kg$^{-1}$ for 5 sequential days or 20 mg kg$^{-1}$ for two courses of 5 days with a 2-day interval. All administrations were given in a volume of 0.01 ml g$^{-1}$ body weight and controls received a corresponding volume of PBS.

Mice were withheld from food 10–12 h before single treatment and 5 h before the daily administration. Water was available ad libitum.

### Biodistribution and bioavailability

[$^{125}$I]MIBG (specific activity 740–925 MBq mg$^{-1}$ was synthesized as described previously (Wieland et al, 1980) and prepared to a specific activity of 0.27 MBq mg$^{-1}$ by addition of unlabelled MIBG for i.v. or p.o. treatment with 15 mg kg$^{-1}$ (0.11 MBq mouse$^{-1}$). After 10 and 30 min and 1, 4 and 24 h, blood was taken from the carotid artery under ether anaesthesia and mice were sacrificed. At each time point, four animals were used. Because MIBG is, at least in humans, metabolized to only a minor degree (Wafelman et al. 1994), [$^{125}$I]MIBG levels could be determined by gamma-counting of 100 μl of blood, 100 μl of plasma, the kidneys, liver and about 2 cm of the small intestine (at 1 cm distance of the stomach and flushed by PBS), which were expressed as percentage of the injected dose per ml of plasma or gram tissue. MIBG retained by the total body was calculated by adding the counts of the remaining body, corrected by 10/9 for differences in counting efficiency, to the counts of the excised tissues and expressed as percentage of total dose. Areas under the concentration–time curve (AUCs) were calculated by the trapezoidal rule with extrapolation to infinity and bioavailability in plasma by the formula AUC p.o./AUC i.v. × 100%. The mean residence time (MRT) was calculated by dividing

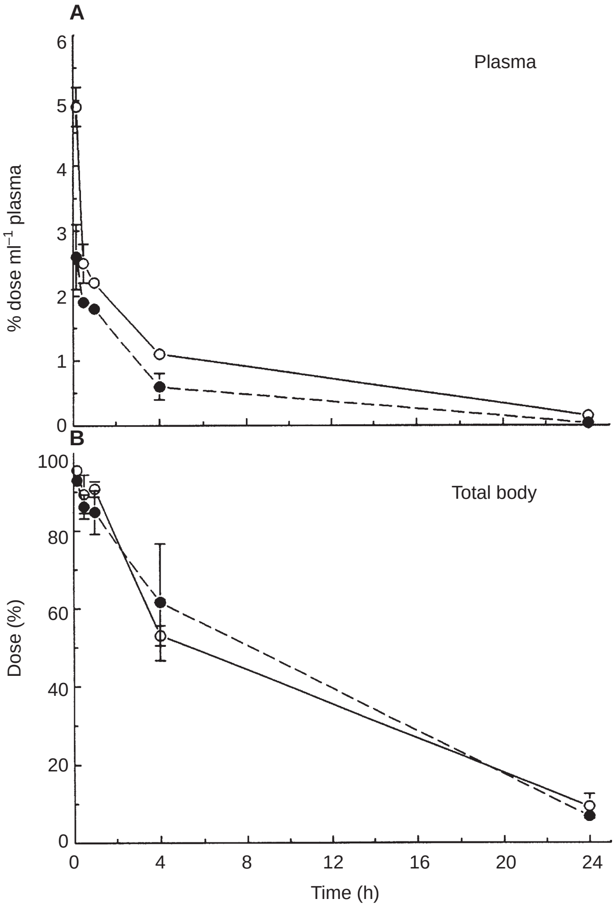

**Figure 1** [$^{125}$I]MIBG levels in plasma (**A**) and total body (**B**) after i.v. (○) or oral (●) administration of 15 mg kg$^{-1}$. Mean values ± s.d. ($n$ = 4). Error bars if not shown are within the size of the symbols the area under the moment curve (AUMC) by the AUC. Clearance (Cl) and volume of distribution ($V_d$) were determined for i.v. administration only by the formulas Cl = dose/AUC and $V_d$ = Cl × MRT respectively. Because plasma concentrations were calculated as % dose ml$^{-1}$ plasma, the AUC was determined as h % dose ml$^{-1}$.

### Renal function

Renal function was measured by determination of the glomerular filtration of [$^{51}$Cr]EDTA as described previously (Kuin et al, 1998). In short, [$^{51}$Cr]EDTA (0.37 MBq 100 μl$^{-1}$, specific activity 37–74 MBq mg$^{-1}$; Amersham International, UK) was injected 4.5 h after single dose MIBG or 1 day after repeated MIBG administration. After 30 min, blood samples were taken from the retro-orbital sinus and the mice were sacrificed. Plasma [$^{51}$Cr]EDTA levels were determined by gamma-counting and expressed as percentage of injected dose per ml plasma. Increases in residual plasma [$^{51}$Cr]EDTA levels above control values reflect reduced renal clearance.

### Toxicity and histological examination

After oral injection of MIBG, mice were weighed daily and checked for visible signs of illness. Mice were sacrificed at 5 h, 24 h and 7, 14 and 48 days after single dose, and 1 day after the

© Cancer Research Campaign 1999

**Table 1** Relative exposure to orally administered MIBG (15 mg kg$^{-1}$)

| Tissue | AUC p.o./AUC i.v. × 100% |
|---|---|
| Plasma | 59 |
| Blood | 63 |
| Kidney | 79 |
| Liver | 123 |
| Small intestine | 165 |
| Total body | 108 |

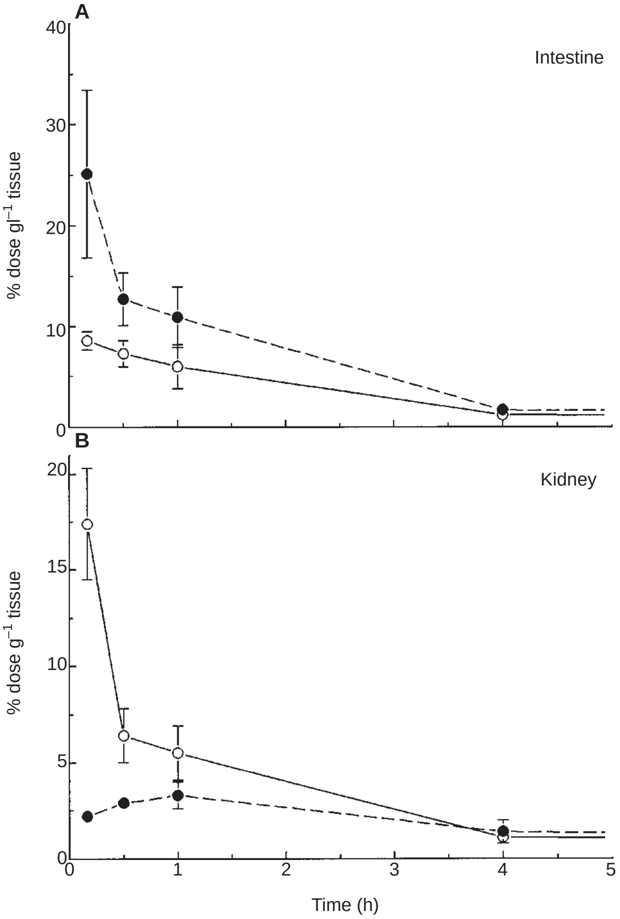

**Figure 2** [$^{125}$I]MIBG levels in small intestine (**A**) and kidney (**B**) during the first 4 h after i.v. (○) or oral (●) administration of 15 mg kg$^{-1}$. Mean values ± s.d. ($n$ = 4). Error bars if not shown are within the size of the symbols last dose of the repeated administration. Dissected organs were routinely processed for histological examination by an animal pathologist.

### RESULTS

### Biodistribution

The bioavailability of orally administered MIBG was determined at 15 mg kg$^{-1}$, which is near to the maximal tolerated dose of i.v.-administered MIBG in C3H/Km mice. Based on the AUCs presented in Figure 1 A, the bioavailability of orally administered MIBG was calculated to be 59%. Based on i.v. MIBG administration the clearance (Cl) was calculated to be 178 ml h$^{-1}$ kg$^{-1}$, the $V_d$ 43.6 ml and the MRT 8.9 h. After oral administration the MRT was 5.8 h. The AUC of the total body was the same for both administration routes (Figure 1 B). Tissue levels after orally administered MIBG were higher for liver and small intestine but lower for the kidney as compared with i.v. administration (Table 1). These differences were observed during the first 4 h only, as illustrated for small intestine and kidney in Figure 2.

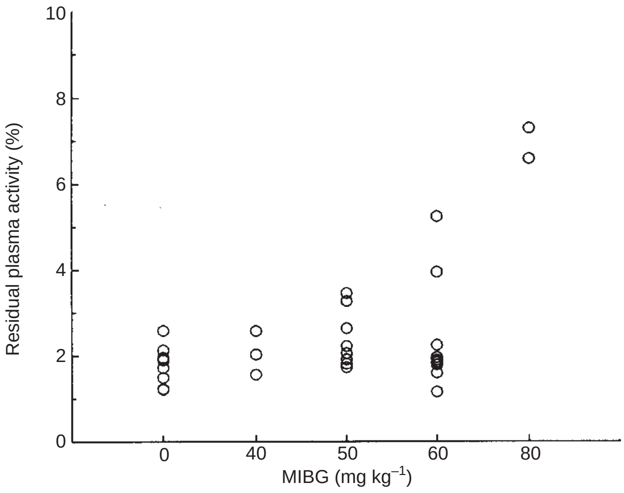

**Figure 3** Renal clearance expressed by residual [$^{51}$Cr]EDTA plasma levels 5 h after single oral MIBG treatment. Controls received a corresponding volume of PBS. Symbols represent results of individual mice in three independent experiments

### Toxicity of a single dose MIBG

MIBG could be given orally up to 60 mg kg$^{-1}$ without lethality. Although at 50 and 60 mg kg$^{-1}$ mean values are not significantly different from controls ($P$ > 0.05) some animals demonstrated a decreased renal clearance (Figure 3). MIBG (80 mg kg$^{-1}$) severely impaired renal clearance in two mice and was acutely lethal to a third.

Renal damage was observed by histological analysis 5 and 24 h after treatment with 50 and 60 mg kg$^{-1}$ and had recovered after 7 days, similar to what was described for i.p.-administered MIBG (Kuin et al, 1998). The damage was observed as acute degeneration and vacuolization of renal epithelial cells of the cortico-medullar tubules, dilatation, most prominent in the distal tubuli, and minor hypertrophy of the epithelium of Bowman's capsule. No histological abnormalities were observed in stomach, intestines, liver, pancreas, bladder, spleen, heart, lungs, thymus, salivary glands, brain and testes up to 48 days after treatment. Also, body weights of treated mice were not different from PBS-treated controls and no visible signs of illness were observed.

### Renal function and toxicity after repeated oral administration

MIBG was administered at 40 mg kg$^{-1}$ over 5 sequential days or at 20 mg kg$^{-1}$ in two courses of 5 days with a 2-day interval. Neither schedule affected body weight in comparison with PBS-treated controls. No visible signs of illness were observed, but mice reacted more alertly and were slightly more stressed than PBS-treated

 © Cancer Research Campaign 1999

mice. No change in renal clearance was observed 1 day after the last treatment. Residual [$^{51}$Cr]EDTA levels per ml plasma were 2.5 ± 0.3% for controls and 2.1 ± 0.4% for 40 mg kg$^{-1}$ MIBG, and 2.2 ± 0.6% for controls and 1.6 ± 0.5% for 20 mg kg$^{-1}$ MIBG, at days 5 and 12 respectively ($n$ = 6).

Parallel histological examination revealed the absence of renal damage and of abnormalities in stomach, intestines, liver, heart, lungs, thymus, salivary glands and testes. In the spleen, hyperaemia of the red pulp was observed after both protocols, which was not observed after a single dose of 60 mg kg$^{-1}$.

### DISCUSSION

Non-radiolabelled MIBG has increasing clinical potential (Taal et al, 1996*a*). In the palliation of the carcinoid syndrome MIBG is effective as a single dose but might require chronic drug administration for maintenance. For this purpose we investigated the feasibility of oral administration. We demonstrated a bioavailability of 59% after oral administration in mice. The absorption of MIBG was fast and highest plasma levels were observed only 5 min after oral administration, indicating that P-glycoprotein-mediated export of MIBG into the intestinal lumen is of minor importance. Plasma and total body MIBG clearance rates were similar for both administration routes (Figure 1). As a result of these similar clearance rates and the absence of a slow-release effect after oral administration, the MRT of MIBG is shorter after oral administration than after i.v. injection. The oral administration routes caused a shift in the biodistribution, mainly into the intestine and liver (Figure 2 and Table 1), without affecting total body clearance. This, however, does not reflect first-pass metabolism as MIBG is metabolized only to a minor degree (Wafelman et al, 1994). Despite a relatively high exposure to the small intestine and liver, no ulceration or other histological damage were observed in these tissues or in the stomach at the MTD. Because of the adequate bioavailability and absence of route-related toxicity, we consider MIBG a suitable compound for further clinical research of the oral administration route.

Side-effects of oral MIBG administration, encountered at ≥ 50 mg kg$^{-1}$, were transient histological tubular damage accompanied in some mice by a reduced renal clearance (Figure 3). These results were similar to those of previous studies demonstrating renal toxicity after i.p. administration of high-dose MIBG, most probably because of the direct toxic effects of MIBG, present in high concentrations in the primary urine (Kuin et al, 1998). In contrast, repeated oral administration of MIBG up to accumulated doses of 200 mg kg$^{-1}$ did not induce histological damage of the kidney or other tissues. The absence of cumulative effects agrees with a previous study demonstrating absence of acute toxicity after repeated treatment at the maximum tolerated dose (Smets et al, 1988), and may be attributed to rapid clearance of the drug (Figure 1B). Hyperaemia of the spleen is probably due to vascular effects as a result of MIBG-induced bioamine release (Kuin et al, 1994) or vasodilatation by inhibition of NOS (Kuin et al, 1998).

The maximal tolerated dose of a single dose of 60 mg kg$^{-1}$ for orally administered MIBG was 3–4 times higher than that for bolus i.v. administration. At the maximal tolerated doses the plasma AUC of MIBG will therefore be higher for the oral than for the i.v. administration. Assuming a dose–response relationship, oral application might consequently increase the response rate in patients with carcinoid syndrome. MIBG was injected i.v. in relative low doses to prevent acute effects of high peak levels. In patients, these acute effects are partly prevented by slow infusion, which will reduce the gain in AUC after oral administration compared with the animal studies. On the other hand, because carcinoid tumours and their metastases are mainly located in the intestines and liver, the relatively high levels of MIBG in these tissues after oral administration might be an additional advantage in targeting these tumour localizations.

In carcinoid patients, side-effects of i.v.-administered MIBG are encountered at a dose of 34 mg m$^{-2}$ (Taal et al, 1996*a*), much lower than that observed in a phase I trial in patients with non-carcinoid tumours (135 mg m$^{-2}$, B. Taal, personal communication). This difference is most probably attributable to a MIBG-induced release of vasoactive compounds, such as serotonin, stored by the carcinoid tumour. Although it could be argued therefore that dose escalation is not a primary goal for improved response rates, our previous study has demonstrated that MIBG-induced vasoactive effects can be antagonized by the 5-HT receptor blocker cyproheptadine (Kuin et al, 1998).

In conclusion, repeated oral administration of MIBG is a promising alternative for long-term palliation of the carcinoid syndrome without the need for hospitalization.

### ACKNOWLEDGEMENTS

The authors gratefully acknowledge P Jonkergouw (Department of Safety and Radiation Protection, The Netherlands Cancer Institute) for [$^{125}$I]MIBG synthesis and Dr B Taal (Department of Gastroenterology, The Netherlands Cancer Institute) for helpful suggestions and critical reading of the manuscript.

### ABBREVIATIONS

AUC, area under the concentration–time curve; AUMC, area under the moment curve; Cl, clearance; MIBG, *meta*-iodobenzylguanidine; MRT, mean residence time; NOS, nitric oxide synthase; PBS, phosphate-buffered saline; s.d., standard deviation; $V_d$, volume of distribution

### REFERENCES

Hoefnagel CA, Voûte PA, de Kraker J and Marcuse HR (1987*a*) Radionuclide diagnosis and therapy of neural crest tumors using iodine-131 metaiodobenzylguanidine. *J Nucl Med* **28**: 308–314

Hoefnagel CA, den Hartog Jager FCA, Taal BG, Abeling NGGM and Engelsman EE (1987*b*) The role of I-131-MIBG in the diagnosis and therapy of carcinoids. *Eur J Nucl Med* **13**: 187–191

Hoefnagel CA, Taal BG, Sivro F, Boot H and Valdés Olmos RA (1998) Enhancement of I-131 MIBG uptake in carcinoid tumors by administration of unlabelled MIBG. *Hybridoma* (in press).

Jaques S, Tobes MC and Sisson JC (1987) Sodium dependency of uptake of norepinephrine and *m*-iodobenzylguanidine into cultured human pheochromocytoma cells: evidence for uptake-one. *Cancer Res* **47**: 3920–3928

Kuin A, Smets LA, Volk T, Paans A, Adams G, Atema A, Jähde E, Maas A, Rajewsky MF, Visser G and Wood P (1994) Reduction of intratumoral pH by the mitochondrial inhibitor *m*-iodobenzylguanidine and moderate hyperglycemia. *Cancer Res* **54**: 3785–3792

Kuin A, Aalders M, van der Valk MA, Frey A, Schmidt HHHW and Smets LA (1998) Renal toxicity of the neuron-blocking and mitochondriotropic agent *m*-iodobenzylguanidine (MIBG). *Cancer Chemother Pharmacol* **42**: 37–45

Loesberg C, van Rooij H, Nooijen WJ, Meijer AJ and Smets LA (1990*a*) Impaired mitochondrial respiration and stimulated glycolysis by *m*-iodobenzylguanidine (MIBG). *Int J Cancer* **46**: 276–281

Loesberg C, van Rooij H and Smets LA (1990*b*) Meta-iodobenzylguanidine (MIBG), a novel high-affinity substrate for cholera toxin that interferes with cellular mono(ADP-ribosylation). *Biochem Biophys Acta* **1037**: 92–99

© Cancer Research Campaign 1999 

Rutgers M, Tytgat GAM, Verwijs-Janssen M, Buitenhuis C, Voûte PA and Smets LA (1993) Uptake of the neuron-blocking agent *meta*-iodobenzylguanidine and serotonin by human platelets and neuro-adrenergic tumour cells. *Int J Cancer* **54**: 290–295

Sisson JC and Wieland DM (1986) Radiolabeled *meta*-iodobenzylguanidine: pharmacology and clinical studies. *Am J Physiol Imaging* **1**: 96–103

Smets LA, Bout B and Wisse J (1988) Cytotoxic and antitumor effects of the norepinephrine analogue meta-iodo-benzylguanidine (MIBG). *Cancer Chemother Pharmacol* **21**: 9–13

Smets LA, Janssen M, Rutgers M, Ritzen K and Buitenhuis C (1991) Pharmacokinetics and intracellular distribution of the tumor-targeted radiopharmaceutical *m*-iodo-benzylguanidine in SK-N-SH neuroblastoma and PC-12 pheochromocytoma cells. *Int J Cancer* **48**: 609–615

Sparreboom A, van Asperen J, Mayer U, Schinkel AH, Smit JW, Meijer DKF, Borst P, Nooijen WJ, Beijnen JH and van Tellingen O (1997) Limited oral bioavailability and active epithelial excretion of paclitaxel (Taxol) caused by P-glycoprotein in the intestine. *Proc Natl Acad Sci USA* **94**: 2031–2035

Taal BG, Hoefnagel CA, Valdes Olmos RA, Boot H and Beijnen JH (1996*a*) Palliative effect of metaiodobenzylguanidine in metastatic carcinoid tumors. *J Clin Oncol* **14**: 1829–1838

Taal BG, Hoefnagel CA, Valdes Olmos RA and Boot JH (1996*b*) The effect of unlabelled MIBG prior to $^{131}$I-MIBG scanning and its potential benefit in the treatment of metastatic carcinoid. *Eur J Gastroent Hep* **8**: A36 (abstract)

Wafelman AR, Hoefnagel CA, Maes RAA and Beijnen JH (1994) Radioiodinated *meta*-iodobenzylguanidine: a review of its biodistribution and pharmacokinetics, drug interactions, cytotoxicity and dosimetry. *Eur J Nucl Med* **21**: 545–559

Wieland DM, Wu JI, Brown LE, Mangner TJ, Swanson DP and Beierwaltes WH (1980) Radiolabeled adrenergic neuron-blocking agents: adrenomedullary imaging with [$^{131}$I]iodobenzylguanidine. *J Nucl Med* **21**: 349–353

 *© Cancer Research Campaign 1999*
